# EhMAPK, the Mitogen-Activated Protein Kinase from *Entamoeba histolytica* Is Associated with Cell Survival

**DOI:** 10.1371/journal.pone.0013291

**Published:** 2010-10-08

**Authors:** Anupama Sardar Ghosh, Doel Ray, Suman Dutta, Sanghamitra Raha

**Affiliations:** Crystallography and Molecular Biology Division, Saha Institute of Nuclear physics, Kolkata, India; INSERM U1016, Institut Cochin, France

## Abstract

Mitogen Activated Protein Kinases (MAPKs) are a class of serine/threonine kinases that regulate a number of different cellular activities including cell proliferation, differentiation, survival and even death. The pathogen *Entamoeba histolytica* possess a single homologue of a typical MAPK gene (EhMAPK) whose identification was previously reported by us but its functional implications remained unexplored. EhMAPK, the only mitogen-activated protein kinase from the parasitic protist *Entamoeba histolytica* with Threonine-X-Tyrosine (TXY) phosphorylation motif was cloned, expressed in *E. coli* and functionally characterized under different stress conditions. The expression profile of EhMAPK at the protein and mRNA level remained similar among untreated, heat shocked and hydrogen peroxide-treated samples in all cases of dose and time. But a significant difference was obtained in the phosphorylation status of the protein in response to different stresses. Heat shock at 43°C or 0.5 mM H_2_O_2_ treatment enhanced the phosphorylation status of EhMAPK and augmented the kinase activity of the protein whereas 2.0 mM H_2_O_2_ treatment induced dephosphorylation of EhMAPK and loss of kinase activity. 2.0 mM H_2_O_2_ treatment reduced parasite viability significantly but heat shock and 0.5 mM H_2_O_2_ treatment failed to adversely affect *E. histolytica* viability. Therefore, a distinct possibility that activation of EhMAPK is associated with stress survival in *E. histolytica* is seen. Our study also gives a glimpse of the regulatory mechanism of the protein under in vivo conditions. Since the parasite genome lacks any typical homologue of mammalian MEK, the dual specificity kinases which are the upstream activators of MAPK, indications of the existence of some alternate regulatory mechanisms of the EhMAPK activity is perceived. These may include the autophosphorylation activity of the protein itself in combination with some upstream phosphatases which are not yet identified.

## Introduction

Mitogen Activated Protein Kinases (MAPK) are a group of proline directed serine/threonine kinases [1] that regulate a number of different cellular processes like cell growth, proliferation, differentiation and response to a variety of environmental stresses like osmotic stress, ultraviolet light, heat shock and hypoxia [2]. In mammals five different sub-groups of MAPK have been reported: the extracellular signal-regulated kinase protein homologues 1 and 2 (ERK 1/2); the big MAPK-1 (BMK-1) also known as ERK5; c-Jun N-terminal kinase homologues 1, 2 and 3 (JNK 1/2/3); the stress activated protein kinase 2 homologues α, β and δ also known as p38α/β/δ and ERK6 also known as p38γ [3]. Each of these MAPK homologues function in a module comprising two more upstream kinase families, MAPK kinase (MEK/MAPKK) which is a serine/threonine and tyrosine dual kinase that activates MAPK, and MAPKK kinase (MAPKKK) which is a serine/threonine kinase that activates MAPKK in response to an external stimuli through a membrane receptor. However till date only three MAPK cascades have been well characterized. These include the ERK1/2 cascade which has been mostly associated with cell growth, proliferation and survival, the JNK signaling cascade that is activated by cellular stresses and the p38 pathway that is activated by a number of inflammatory cytokines as well as pathogens and environmental stresses [2]. In parasitic protozoa the existence of MAPK homologues has been documented. It has also been shown that MAPK homologues exhibit differential activities in response to a wide variety of stimuli. For instance ERK1 and ERK2 homologues of *Giardia lamblia* have been shown to play a critical role in trophozoite differentiation into cysts [4], Pbmap2, a MAPK homologue in *Plasmodium berghei* has been shown to be essential for male gametogenesis in the mosquito vector [5], Pfmap2, a MAPK homologue in *Plasmodium falciparum* is essential for the completion of the asexual phase of the parasite lifecycle [6] and *Leishmania major* MAPK homologues exhibit an increased phosphotransferase activity in response to pH and temperature shift [7].

*E. histolytica*, the causative agent of human amoebiasis is a microaerophilic parasitic protozoan that normally resides within the human gut where it is exposed to a number of different stresses. Moreover outside the host it faces even harsher environmental constraints that often lead to the formation of resistant cystic forms of the parasite. Variation in gene expression patterns have been observed in *E. histolytica* in oxidative and nitrosative stresses [8]. In the present study we report the differential activation of EhMAPK, a MAPK homologue from *E. histolytica* in response to heat shock at 43°C and oxidative stress induced by treatment with H_2_O_2_. This particular MAPK homologue has previously been identified and characterized in our lab and its detailed sequence analysis together with the phylogenetic study placed it in the ERK sub-family of MAPK with high sequence homology to mammalian ERK7/8 [9]. A later study of the kinome of the parasite revealed the presence of another atypical MAPK homologue in the genome that unlike the previous one lacked a conserved TXY phosphorylation motif [10]. Moreover the absence of an upstream MEK/MAPKK from the parasite genome [10] indicates a probable MAPKK-independent pathway of MAPK activation in *E. histolytica*. Our present data strongly suggests a possible role of EhMAPK activity in promoting survival of the parasite under conditions of stress.

## Methods

### Cell Culture

*E. histolytica* trophozoites were cultured axenically at 37°C in TYI-S-33 [11] medium supplemented with antibiotics benzylpenicillin and streptomycin. Cells were sub-cultured every 72–96 h and harvested by centrifugation at 300 g for 5 min followed by two washes with phosphate buffer saline, PBS (140 mM NaCl, 2.7 mM KCl, 10 mM KH_2_PO_4_/K_2_HPO_4_, pH: 7.5) for 5 min each.

For the purpose of cloning and expression of recombinant EhMAPK, *E. coli* DH10B and *E. coli* BL21 DE3 strains were used respectively which were grown in Luria Bertani (LB) medium supplemented with kanamycin at a final concentration of 30 µg/ml when required.

### Genomic DNA preparation

The genomic DNA from *E. histolytica* trophozoites was isolated using a protocol adapted from Wilson et al [12] with some minor modifications. Briefly about 1×10^6^ trophozoites were suspended in 500 µl of PBS containing 10 µg/ml RNase A, 50 µg/ml proteinase K and 0.5% w/v SDS with a subsequent incubation at 65°C for 15 min followed by overnight incubation at 37°C. DNA was then extracted thrice with phenol-chloroform and finally precipitated using ethanol. 10 µg of glycogen was then added to the precipitating DNA solution which was incubated at −20°C for 45 min. The precipitated DNA was then washed with 70% v/v ethanol, resuspended in TE buffer (10 mM Tris-HCl, 1 mM EDTA, pH: 8.0) and kept at −20°C in small aliquots until further use.

### Ligation-Independent Cloning of EhMAPK gene

The EhMAPK gene (Gen- Bank™ accession number AY460178) from *E. histolytica* genomic DNA was PCR amplified as described in [9] using the primers MAPKfwd and MAPKrev (sequences are enlisted in Table1) and cloned in pET30 Ek/LIC vector using the Ligation-Independent Cloning kit from Novagen following manufacturer's protocol. 100 ng of the annealed product was electro-transformed in electro-competent *E. coli* DH10B cells in a Biorad Gene Pulser at 2500 V, 25 µF and 200 ω which were then grown in LB medium at 37°C for 1 h followed by spreading on LB agar plates supplemented with 30 µg/ml of kanamycin. The plasmids from the colonies that were found to be positive for the clone were confirmed by sequencing (Bangalore Genei) and then used to electro-transform *E. coli* BL21 DE3 cells for recombinant protein expression.

**Table 1:** Sequences of the primers used in this study.

Primers	Sequences
MAPKfwd	(5′) GACGACGACAAGATGAGTGATAAAGAGTATATG (3′)
MAPKrev	(5′) GAGGAGAAGCCCGGTTTATTTCAACATATAATGA (3′)
ExMAPKfwd	(5′) ACTGACCTTCATGCAGTTATTAG (3′)
ExMAPKrev	(5′) CTTTTTCTGCTGTTGCACGTTT (3′)
Eactfwd	(5′) CTTGTTGTAGATAATGGAT (3′)
Eactrev	(5′) CTGAGTATTTTCTTTCTG (3′)
T178Afwd	(5′) AACTCTTCAAGCAGATTACG (3′)
T178Arev	(5′) CGTAATCTGCTTGAAGAGTT (3′)
Y180Afwd	(5′) CAAACAGATGCCGTTGAAAC (3′)
Y180Arev	(5′) GTTTCAACGGCATCTGTTTG (3′)

### Site directed mutagenesis of EhMAPK gene and subsequent cloning in pET30 Ek/LIC vector

Mutagenesis of the EhMAPK gene was performed by PCR in two steps. First two DNA fragments EhMAPK1 and EhMAPK2 were obtained from the genomic DNA of *E. histolytica* trophozoites through PCR with a pair of mutagenic oligonucleotides in combination with the oligonucleotide primers MAPKfwd and MAPKrev that are complementary to the 5′ and the 3′ end of the EhMAPK gene respectively. The mutagenic oligonucleotide pairs T178Afwd, T178Arev and Y180Afwd, Y180Arev were designed to substitute threonine and tyrosine residues respectively within the TXY activation motif of EhMAPK gene with alanine. The resulting PCR amplified mutant genes were then cloned within the ligase-independent cloning site of pET30 Ek/LIC vector as mentioned in the previous section. The sequences of the mutagenic oligonucleotide primer pairs are listed within table 1.

### Purification of recombinant wild type and mutant EhMAPK proteins and antibody production

500 ml of LB medium was inoculated with 5 ml of an overnight culture of transformed *E. coli* BL21 DE3 overexpressing either the mutant or the wild type EhMAPK protein and grown at 37°C until OD_600_ reached 0.6. Recombinant protein expression was then induced by 1 mM IPTG and the culture was further grown at 37°C for 3 h after which the cells were harvested by centrifugation at 4500 g for 15 min at 4°C and stored at −20°C till further use. To purify inclusion body the cell pellet was resuspended in 5 ml of lysis buffer containing 50 mM Tris, 300 mM NaCl, pH 8.0 supplemented with 1 mg/ml lysozyme, 1 X protease inhibitor cocktail (Pierce) and 5 mM beta mercaptoethanol and incubated on ice for 30 min followed by sonication till a fairly clear non-viscous lysate was obtained. The lysate was centrifuged at 16000 g for 30 min and the pellet was dissolved in 5 ml of inclusion body solubilizing buffer (50 mM Tris, 300 mM NaCl, 8 M urea, 10 mM imidazole, pH 8.0). After 2 h incubation at room temperature the suspension was centrifuged at 16000 g for 30 min to remove any insoluble material. The solubilised inclusion body fraction was then loaded on a pre equilibrated Ni-NTA column (Qiagen) and washed with 15 ml of inclusion body solubilizing buffer supplemented with 25 mM imidazole. The affinity bound recombinant protein was then eluted with 1 ml of 500 mM imidazole in inclusion body solubilizing buffer. For antibody production the eluted wild type protein was subjected to sodium dodecyl sulfate polyacrylamide electrophoresis (SDS-PAGE) and coomassie staining. After destaining the recombinant EhMAPK band was excised from gel and about 3 mg was sent for antibody production in New Zealand rabbits (Imgenex India).

### Refolding of the purified recombinant EhMAPK

For refolding of the purified recombinant EhMAPK the eluted portions were diluted to a final concentration of 5 µg/ml in the refolding buffer (50 mM Tris pH 8.0, 150 mM NaCl, 15% glycerol, 10 mM reduced glutathione and 1 mM oxidized glutathione) at 4°C. The refolded protein was then concentrated using 30 KDa centricon to a final concentration of 2 µg/µl and stored at 4°C until further use.

### Treatment of *E. histolytica* trophozoite cultures

The *E. histolytica* trophozoites from 48 h cultures were subjected to two different stresses, heat shock at 43°C and exposure to either 2.0 mM or 0.5 mM H_2_O_2_. For heat shock experiments the culture tubes were placed in a water bath at 43°C. H_2_O_2_ treatment was however carried out at 37°C incubator in serum deprived media.

### Trypan Blue viability assay

Effect of H_2_O_2_ and heat shock on the viabilities of *E. histolytica* trophozoites was assessed by staining both the treated and untreated control cells with 0.2% trypan blue stain (GIBCO, Invitrogen Corporation). The trophozoites were incubated with either 0.5 mM or 2.0 mM H_2_O_2_ for a total period of 3 h and viability was assessed after every 20 min and compared with that of the untreated control cells. Similarly for heat shock experiment the trophozoites were incubated in a water bath at 43°C for a period of 3 h and viability was assessed every after 20 min.

### Reverse Transcriptase Polymerase Chain Reaction (RT-PCR)

Total RNA from either untreated control or treated trophozoites was isolated using TRIZOL reagent (Ambion) following manufacturer's protocol. For first strand cDNA synthesis 2 µg of total RNA from each sample was reverse transcribed with oligo dT primer using 0.2 mM dNTP mix, 25 U RNase inhibitor and 100 U Moloney Murine Leukemia Virus Reverse Transcriptase. Subsequent PCR was then carried out with 2 µl of cDNA in a 25 µl reaction mix containing 10 pmol of each of the gene specific forward and reverse primers, 250 µM of dNTPs, 5 U of taq polymerase in a buffer containing 1.5 mM MgCl_2_. The PCRs were initially standardized for the number of cycles that yield products in the logarithmic phase before saturation was reached. In case of both actin and MAPK, saturation was reached at and after 31 cycles in all the samples analysed. So, the respective PCRs were carried out for 27 cycles that was well below the saturation limit.

The sequences of the primers used for the amplification of EhMAPK cDNA, ExMAPKfwd and ExMAPKrev and *E. histolytica* beta actin cDNA, Eactfwd and Eactrev are listed in table1. Beta actin PCR product was used as a loading control.

### *E. histolytica* whole cell protein preparation

The cell pellet from 48 h cultures of either untreated control or treated *E. histolytica* trophozoites were suspended in 400 µl lysis buffer (50 mM Tris-HCl, pH 7.4, 5 mM EDTA, 50 mM NaF, 1 mM Na_3_VO_4_) supplemented with 0.5% v/v Triton X-100, 1X protease inhibitor cocktail (Pierce), 1 mM PMSF and 1 µM E-64 and kept on ice for 30 min with intermittent vortexing followed by centrifugation at 500 g for 30 min at 4°C for removing the insoluble cellular debris. The supernatant representing the total cell lysate (TCL) was carefully collected and the protein concentration was estimated using Bradford's reagent. For SDS-PAGE analysis the lysate was boiled with 4X Laemmli buffer (250 mM Tris-HCl, pH 6.8, 8% w/v SDS, 40% v/v glycerol and bromophenol blue).

### Immunoprecipitation

60 µl of the 50% protein A agarose slurry (Bangalore Genei) was incubated with 750 µg of the TCL protein from either treated or untreated control *E. histolytica* trophozoites for 2 h at 4°C with gentle agitation. The suspension was centrifuged at 3000 g for 1 min at 4°C and the supernatant representing the pre-cleared lysate was then incubated with rabbit anti-phospho ERK1/2 at a dilution of 1∶50 for 2 h at 4°C again with gentle agitation. The antibody-antigen complex thus formed was bound to the protein A agarose by incubation with 60 µl of 50% protein A agarose slurry for 2 h at 4°C. The agarose beads containing the immune-precipitate was then washed 5 times with the lysis buffer and finally collected by centrifugation. After keeping a small amount of the beads for kinase assay the rest of the beads were suspended in 1X Laemmli buffer and boiled for western blot analysis.

### Kinase assay

Kinase assay was performed either with 8 µl of final immuno-precipitate beads or 2 µg of recombinant EhMAPK in a 15 µl reaction mix containing 50 mM Tris-HCl pH 8.0, 0–30 mM MgCl_2_ (as required), 5 µg Myelin Basic Protein(MBP), 50 µM ATP and 5 µCi of [γ-^32^P] ATP. MBP is the major structural protein of the myelin sheath in the central nervous system and a specific substrate for MAPKs specifically ERK1 and ERK2. It contains the consensus sequence Pro-X-(Ser/Thr)-Pro that is recognized by the MAPKs and is phosphorylated at Threonine97 residue [13]. The reaction was carried out at 25°C for 15 min after which it was stopped by adding 5 µl 4X Laemmli buffer and boiling for 5 min. The samples thus prepared were subjected to 12.5% SDS-PAGE. The coommassie stained gel was dried after proper destaining and the dried gel was exposed to X-ray films at −80°C. For non-radioactive kinase assays however the concentration of cold ATP in the reaction mix was increased to 100 µM and no [γ-^32^P] ATP was used. The assay products were then probed with either anti-phospho tyrosine, anti-phospho serine/threonine, anti-phospho ERK1/2 or anti-EhMAPK antibodies in different western blots.

### Phosphatase treatment

To 60 µg of the TCL from either untreated control or treated *E. histolytica* trophozoites (the composition of the lysis buffer used is the same as for TCL preparation as described in the earlier section except for the Na_3_VO_4_ which being a phosphatase inhibitor is omitted) 400 U of λ phosphatase (NEB) was added in 1X assay buffer and 1X MnCl_2_ for 20 min at 30°C. The reaction was stopped by immediately adding 4X Laemmli buffer and boiling for 5 min. The protein samples thus prepared were then analysed by western immunoblotting. However for determining the phosphorylation status of the TXY motif within the recombinant refolded EhMAPK, either 0.5 µg of PTP1B, a tyrosine specific phosphatase (Cayman chemicals) or 0.25 µg of PP2A, a serine/threonine specific phosphatase (Cayman chemicals) were added to the kinase assay products carried out with 2 µg of the purified protein and incubated for 15 min at 37°C. The reaction was then stopped by immediately adding 4X Laemmli buffer and boiling for 5 min. The protein samples were then probed in several western blots with one of the four antibodies, anti-phospho tyrosine, anti-phospho serine/threonine, anti-phospho ERK1/2 and anti-EhMAPK.

### Western immunoblotting

40 µg TCL or other protein samples prepared as described in the previous sections were separated by SDS-PAGE and transferred onto a PVDF membrane. The membranes were then immersed in blocking buffer which is 5% BSA in 1X TBST (25 mM Tris-HCl, pH 7.5, 150 mM NaCl, 0.05% v/v Tween 20) and kept at room temperature under mild shaking condition for 2 h followed by overnight incubation with either 1∶6000 dilution of rabbit anti-EhMAPK anti sera (pre and post immune), 1∶500 dilution of mouse monoclonal anti-βactin (abcam), 1∶1000 dilution of rabbit anti-phospho ERK1/2 (Cell Signaling Technologies -CST 9101), 1∶1000 dilution of mouse monoclonal anti-His antibody (Santa Cruz), 1∶1500 dilution of rabbit anti-phospho tyrosine (abcam) or 1∶2500 dilution of mouse anti-phospho serine/threonine (BD Biosciences) as required. The anti-phospho ERK1/2 antibody is raised against the phosphorylated TXY motif of human p44/42and has been found to recognize endogenous levels of human p44 and p42 MAP kinases when phosphorylated either individually or dually. After washing several times with TBST the membranes were incubated with horseradish-peroxidase conjugated secondary antibodies for 2 h at room temperature. The membranes were then washed several times with TBST and developed by chemiluminescence.

## Results

### Purification of recombinant EhMAPK and subsequent characterization of anti EhMAPK antibody

**Figure 1:**
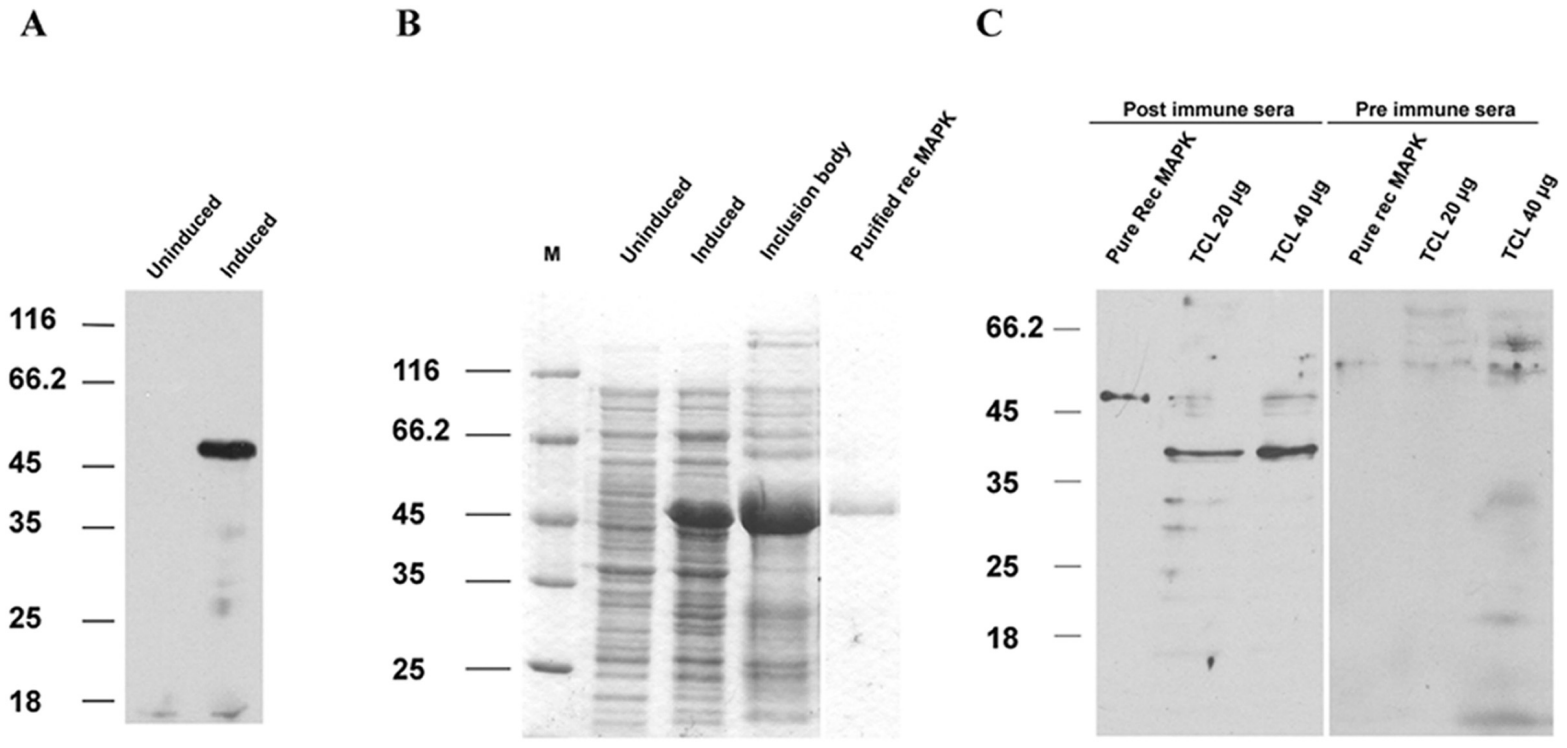
Expression, purification and antibody production of recombinant EhMAPK. (a) Western blot analysis with mouse anti-His antibody to check the expression of recombinant His tagged EhMAPK. Lane1: uninduced *E. coli* BL21DE3 cell lysate, Lane 2: Cell lysate from *E. coli* BL21DE3 induced with 1 mM IPTG at 37°C for 3 h. (b) Coomassie stained gel showing the purification of recombinant His tagged EhMAPK protein with Ni-NTA affinity chromatography. Lane 1: Protein molecular weight marker (Fermentas), Lane 2: Uninduced transformed *E. coli* BL21DE3 cell lysate, Lane 3: Cell lysate from transformed *E. coli* BL21DE3 induced with 1 mM IPTG at 37°C for 3 h, Lane 4: Inclusion body solubilised in 50 mM Tris pH 8, 300 mM NaCl, 8 M urea and 10 mM imidazole, Lane 5: Purified recombinant EhMAPK. (c) Western immunoblotting with post and pre immune sera from rabbits immunized with purified recombinant EhMAPK. Lane 1–3: 1 µg of purified recombinant EhMAPK, 20 µg and 40 µg of *E. histolytica* total cell lysate (TCL) respectively probed with post immune rabbit sera, Lane 4–6: 1 µg of purified recombinant EhMAPK, 20 µg and 40 µg of *E. histolytica* TCL respectively probed with pre immune rabbit sera.

The transformed *E. coli* BL21 DE3 cells containing the EhMAPK clone in pET30 Ek/LIC vector, when induced with 1 mM IPTG for 3 h at 37°C produced the recombinant EhMAPK protein with the 6X His tag. This was further confirmed by western blot analysis using anti 6X His antibody which showed the unambiguous presence of a band migrating at ∼47 KDa in the induced sample only (Fig. 1a). The observed molecular weight also conforms to that theoretically calculated (∼40 KDa + ∼7 KDa vector encoded tag). The subsequent purification of the protein from the inclusion body on a Ni-NTA agarose column yielded about 95% pure recombinant EhMAPK protein (Fig. 1b). Antibody against the purified recombinant EhMAPK was then raised in rabbit. The specificity of the antisera against EhMAPK was analysed by western blot with *E. histolytica* total cell lysate and purified recombinant EhMAPK with both the pre and the post immune sera. The blots displayed bands at the expected molecular weights of ∼40 kDa and ∼47 kDa respectively by the post immune sera but not by the pre immune sera indicating a clear recognition of the endogenous EhMAPK as well as the purified recombinant EhMAPK (Fig. 1c).

### Characterization of the kinase activity of the refolded recombinant EhMAPK

**Figure 2:**
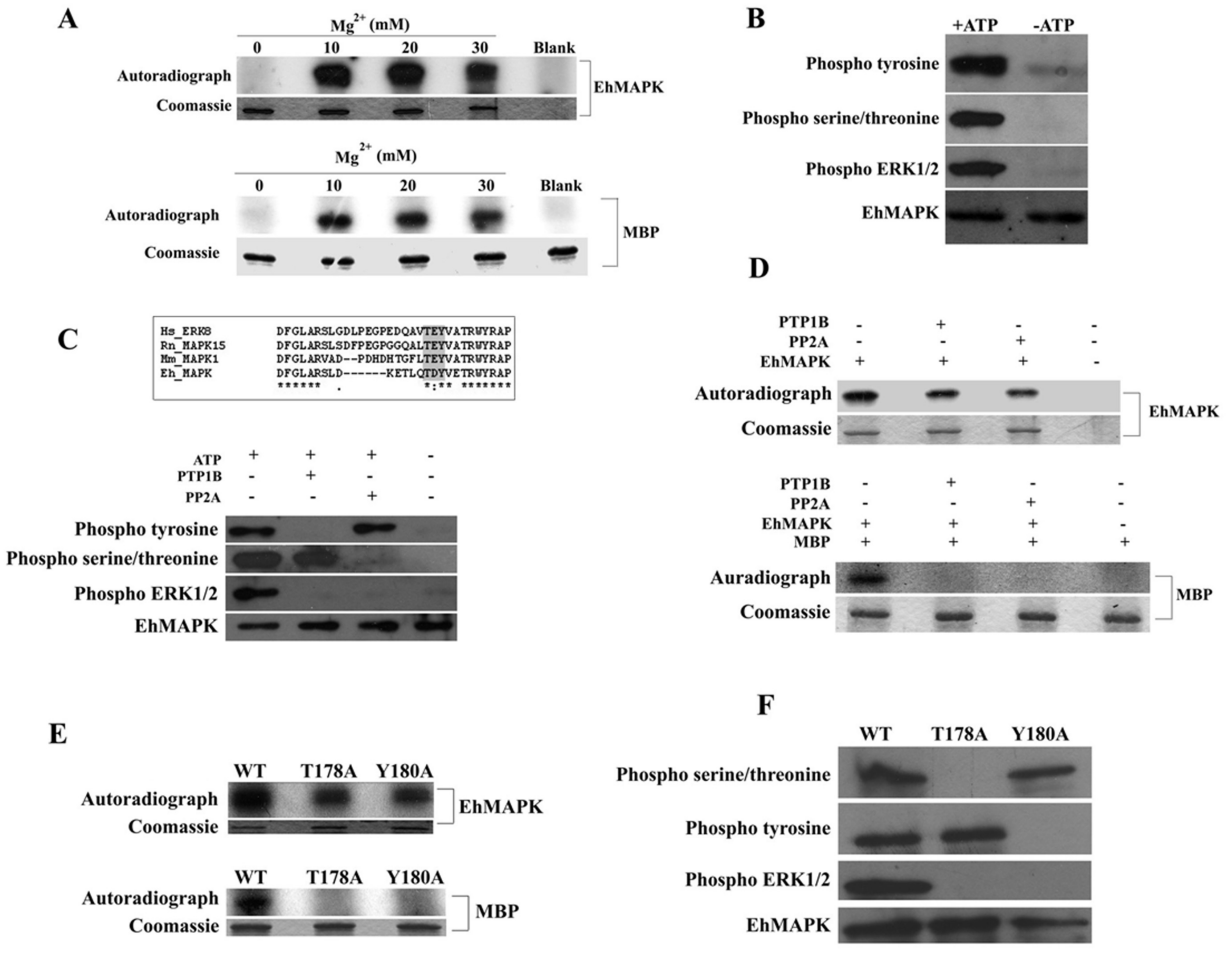
Kinase assay with the recombinant refolded EhMAPK. (a) Mg^2+^ dependence of the kinase activity of EhMAPK, lane 1–4: 0.0 mM to 30.0 mM Mg^2+^, lane 5: Protein blank. Both the EhMAPK (top) and the MBP (bottom) have been shown. In each figure lower panel represents coomassie staining of the respective proteins and upper panel represents the autoradiograph of the corresponding dried gel. (b) Western blot of the EhMAPK kinase assay samples with anti-phospho tyrosine, anti-phospho serine/threonine, anti-phospho ERK1/2 and anti-EhMAPK antibodies respectively from top to bottom, either in the presence (lane 1) or in the absence (lane 2) of ATP. (c) Sequence alighnment of EhMAPK and mammalian MAPKs showing the conservation of the active site sequence; the highlighted region indicate the TXY activation motifs; Hs, Homo sapiens ERK8 (AAl40897); Rn, Rattus norvegicus MAPK15 (NP_775453); Mm, Mus musculus MAPK1 (NP_001033752). Western blot of purified EhMAPK kinase assay samples after treatment with either tyrosine phosphatase, PTP1B or serine/threonine phosphatase, PP2A. The blots were probed with anti-phospho tyrosine, anti-phospho serine/threonine, anti-phospho ERK1/2 and anti-EhMAPK antibodies. Lane 1: in the absence of both PTP1B and PP2A but in the presence of ATP, lane 2: In the presence of PTP1B and ATP but in the absence of PP2A, lane 3: in the presence of PP2A and ATP but in the absence of PTP1B, lane 4: In the absence of ATP, PTP1B and PP2A. (d) Kinase assay in the presence of phosphatases. Both the EhMAPK (top) and MBP (bottom) have been shown. In each figure lower panel represents coomassie staining of the respective proteins and the upper panel represents autoradiograph of the corresponding dried gels. Lane 1: In the absence of either of the phosphatases, lane 2: in the presence of PTP1B, lane 3: in the presence of PP2A, lane 4: EhMAPK Blank. (e) Kinase assay with the wild type and mutant EhMAPKs. Both the EhMAPK (top) and MBP (bottom) has been shown. In each figure lower panel represents coomassie staining of the respective proteins and upper panel represents the autoradiographs of the corresponding dried gels. Lane 1: wild type, lane 2: T178A mutant EhMAPK, lane 3: Y180A mutant EhMAPK. (f) Western blot analysis of the kinase assay samples from wild type and mutant EhMAPKs. The blots were probed with anti-phospho serine/threonine, anti-phospho tyrosine, anti-phospho ERK1/2 and anti EhMAPK (top to bottom), lane 1: wild type, lane 2: T178A mutant EhMAPK, lane 3: Y180A mutant EhMAPK.

Kinase assay with the purified recombinant refolded EhMAPK indicated Mg^2+^-dependent kinase activity of the protein. As evident from Fig. 2a, EhMAPK undergoes autophosphorylation besides being able to phosphorylate MBP, a typical MAPK substrate. Although no significant change in the kinase activity of the protein was observed when Mg^2+^ concentration was varied from 10 mM to 30 mM, the activity was completely abolished in the absence of the divalent cation. So, 10 mM Mg^2+^ was used in all further kinase assay experiments. In order to further characterize the autophosphorylation activity of recombinant EhMAPK kinase, assays were performed in the absence of MBP and both in the presence and absence of ATP. The resulting products were probed with anti-phospho tyrosine, anti-phospho serine/threonine, anti-phospho ERK1/2 and anti-EhMAPK antibodies in several western blots. Fig. 2b shows that recombinant EhMAPK can get autophosphorylated on both serine/threonine as well as tyrosine residues. To confirm that the phosphorylated threonine and tyrosine are those in the TXY activation motif, the autophosphorylated EhMAPK was probed with an anti phospho human p42/44 ERK1/2 antibody. This commercially available antibody has been described to detect endogenous levels of either individually or dually phosphorylated human p44 and p42 MAP kinases phosphorylated at Thr202 and Tyr204 of p44 and Thr185 and Tyr187 of p42. So, it became a necessity in our case to check the specificity of the antibody towards both the singly and the dually phosphorylated forms of EhMAPK.

Although the activation site sequence of EhMAPK is highly homologous with other mammalian MAPKs (Fig. 2c), a considerable deviation in the activation lip containing TXY motif has already been noticed in the atomic model of the protein built on rat ERK2 as template [9]. So, to investigate whether this deviation has any effect on the recognition of the singly phosphorylated forms of the protein by the antibody we carried out kinase assay with the recombinant protein followed by incubation with either PTP1B or PP2A. As PTP1B is a tyrosine specific phosphatase, treatment with this enzyme will in effect generate threonine phosphorylated form of recombinant EhMAPK. Similarly, treatment of the dually phosphorylated recombinant EhMAPK with serine/threonine specific phosphatase PP2A will generate tyrosine phosphorylated form of the protein. A subsequent western blot analysis of the treated samples with antiphospho ERK1/2 will then indicate the specificity of the antibody. Thus the resulting products were probed with one of the four antibodies mentioned above in different western blots. As evident from Fig. 2c unlike untreated kinase assay samples neither of the PTP1B-treated samples nor the PP2A- treated samples were recognized by anti-phospho ERK1/2 antibody indicating the specificity of the antibody to only the dually phosphorylated TXY motif of EhMAPK. On the contrary, the anti EhMAPK antibody recognized both the treated as well as the untreated samples, anti phospho tyrosine recognized the untreated and the PP2A-treated samples and anti phospho serine/threonine antibodies recognized the untreated and the PTP1B-treated samples thereby serving as proper controls of the experiment. To critically evaluate the kinase activity of EhMAPK that is singly phosphorylated at its TXY motif, radioactive kinase assays were performed in the presence of either of the phosphatases. Although in the absence of the phosphatases a significant kinase activity was found both with respect to the EhMAPK itself as well as MBP, in the presence of either of the phosphatases no kinase activity of the protein was found towards its substrate. However the autophosphorylation was still found albeit slightly lower compared to the untreated samples (Fig. 2d). Although this experiment gives a preliminary idea about the relative inactivity of the protein in its singly phosphorylated forms further experiments were needed to establish the fact. Moreover, significant signals obtained from the EhMAPK bands of the kinase assays with either of the two phosphatase treated samples indicate that despite the presence of one of the phosphatases, the phosphorylation event at the unaffected residue within the activation motif was occurring. The intensities when compared to that of the phosphatase-untreated samples were lower which further confirms the dephosphorylation at either threonine or tyrosine residue depending on whichever phosphatase is present in the reaction mixture. But when the intensities of the substrate MBP was compared almost no signal was obtained from the phosphatase-treated samples. Although the data from this experiment is good enough to negate the kinase activity of threonine phosphorylated EhMAPK towards MBP, no conclusion can be drawn for tyrosine phosphorylated form of the protein since a kinase assay in the presence of PP2A will lead to the dephosphorylation of the substrate MBP which is known to be phosphorylated by ERK1/2 at threonine 97 residue [13], in addition to the dephosphorylation at threonine residue within the TXY activation motif of EhMAPK. Thus, to further evaluate the activities of the singly phosphorylated forms of the protein, kinase assays were performed with both the purified T178A and the Y180A mutant EhMAPK proteins. As expected, none of the mutant proteins showed any activity towards MBP when compared with the wild type and the autophosphorylation was also found to be significantly lower than that of the wild type protein (Fig. 2e). A non radioactive kinase assay with the recombinant EhMAPKs in the absence of MBP followed by western blot analysis with either anti-phospho threonine, anti-phospho tyrosine, anti-phospho ERK1/2 or anti EhMAPK antibodies revealed that despite the substitution of one of the phosphorylatable residues within the TXY motif of the mutant EhMAPKs, the phosphorylation at the other residue was still taking place (Fig. 2f). For instance, after kinase assay the T178A mutant although not recognized by anti-phospho threonine was clearly recognized by anti-phoshotyrosine indicating that a phosphorylation event has taken place at the tyrosine residue. Similar results were obtained in case of Y180A mutant the phosphorylated form of which was not recognized by anti-phospho tyrosine but distinctly by anti-phospho threonine antibody. Additionally, neither of the phosphorylated mutant EhMAPKs were recognized by anti-phosphoERK1/2 which again confirmed the specificity of this heterologous antibody towards the dually phosphorylated form of EhMAPK.

### Effect of heat shock and H_2_O_2_ treatment on the transcript levels of EhMAPK gene

**Figure 3:**
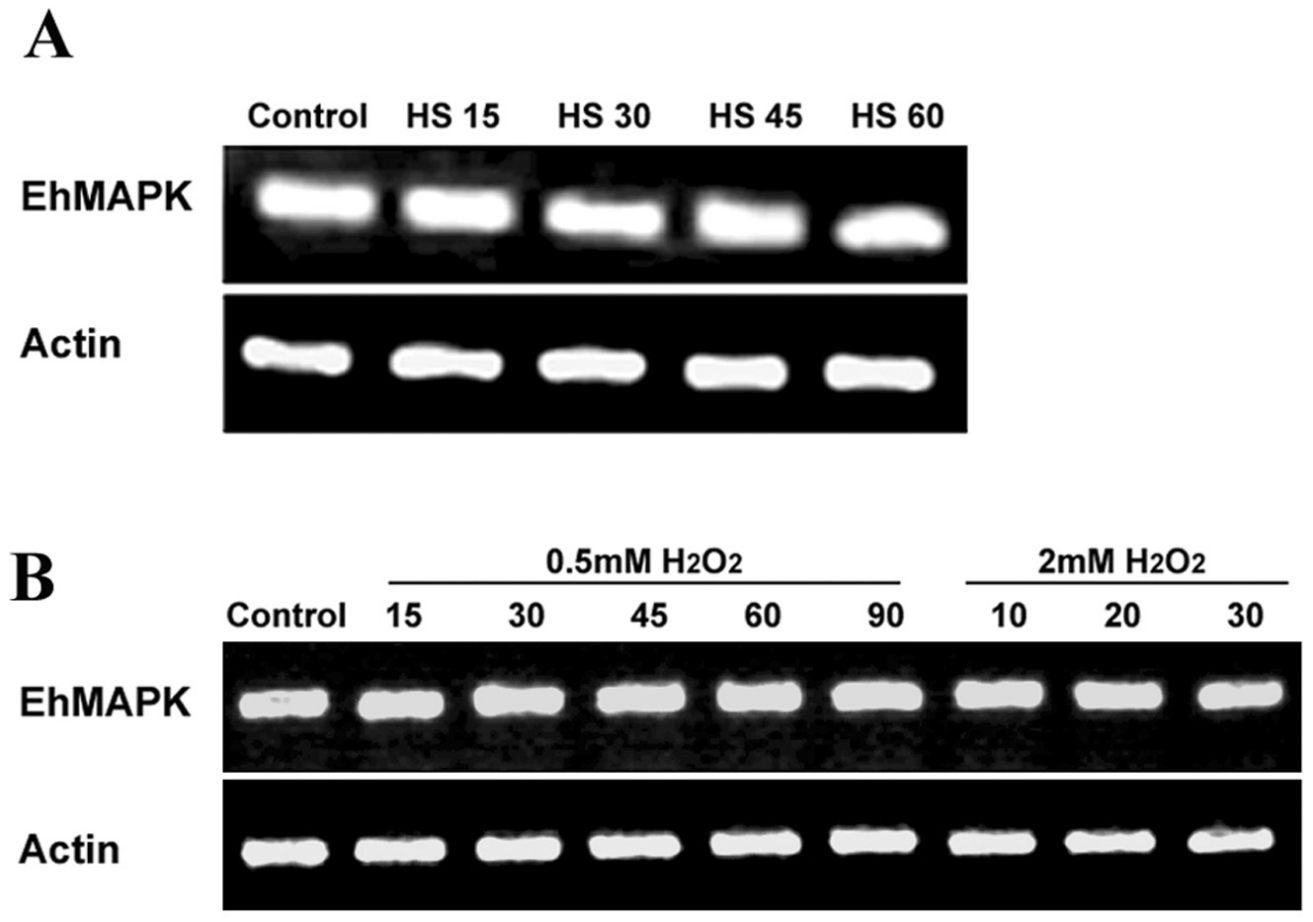
Semi-quantitative RT–PCR showing the transcript levels of EhMAPK gene in H_2_O_2_ treated, heat shocked and untreated *E. histolytica* trophozoites. (a) EhMAPK mRNA levels of *E. histolytica* trophozoites subjected to heat shock at 43°C for different time periods. Lane 1: untreated control trophozoites, Lane 2–5: heat shock at 43°C for 15, 30, 45 and 60 minutes respectively. (b) EhMAPK mRNA levels of *E. histolytica* trophozoites exposed to 0.5 mM or 2.0 mM H_2_O_2_ for different time periods. Lane 1: untreated control trophozoites, Lane 2–6: 0.5 mM H_2_O_2_ treatment for 15, 30, 45, 60 and 90 minutes respectively, Lane 7–9: 2.0 mM H_2_O_2_ treatment for 10, 20 and 30 minutes respectively.

**Figure 4:**
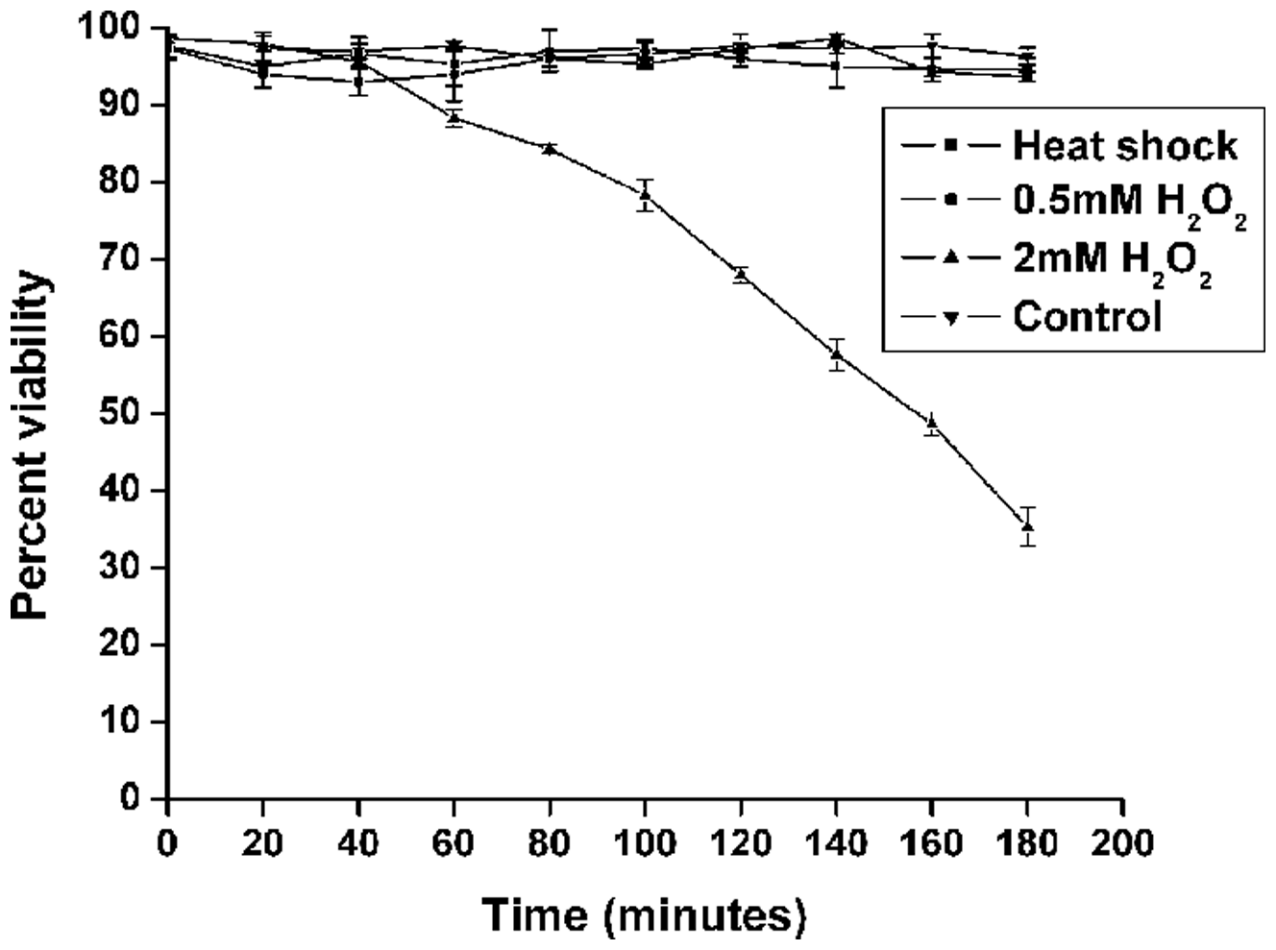
Effect of heat shock and H_2_O_2_ on the viability of *E. histolytica* trophozoites. The vital dye trypan blue was used to discriminate between live and the dead trophozoites. As compared to the untreated control trophozoites (▾) which shows a stable viability curve, 2.0 mM H_2_O_2_ treated trophozoites (▴) however are associated with a gradual loss of viability being about 30% at the end of 3 h incubation period. The viability curve for 0.5 mM H_2_O_2_ treated trophozoites (•) is more or less similar to that of control. Similar is the case with heat shock which also shows a stable viability curve (▪). The viabilities are represented as percentage of the viable cells out of the total population. The data are presented as mean ± SD of the viability percentages from three independent experiments.

The mRNA levels of *E. histolytica* MAPK gene was found to remain unaltered in response to either heat shock or H_2_O_2_ treatment. When compared to control, the transcript levels from the trophozoites exposed to heat shock for up to 60 min at 43°C did not show any significant change as evident from Fig. 3a. Similarly no change in the transcript levels of EhMAPK was noticed in the trophozoites exposed to either 0.5 mM H_2_O_2_ for different time periods upto 90 min or 2.0 mM H_2_O_2_ for upto 30 min (Fig. 3b). We have previously studied the effect of different concentrations of H_2_O_2_ on the viability of *E. histolytica* trophozoites and we found that 2.0 mM H_2_O_2_ can drastically reduce the viability of the trophozoites to about 5% over a time period of 6 h but 0.5 mM H_2_O_2_ can not [14]. In the present study the trypan blue viability assay for control, heat shocked and 0.5 mM H_2_O_2_ treated cells showed little change in viability over a time course of 3 h. But upon treatment with 2.0 mM H_2_O_2_ the viability remained comparable with the other sets for only until 30 min of incubation after which the viability started reducing drastically (Fig. 4). However, after exposure to 2 mM hydrogen peroxide, externalization of phosphatidylserine already became evident at 30 min which was followed later by demonstration of loss of viability and DNA fragmentation at 1h [14]. Hence the 30 min time point was chosen as the optimum time of incubation for further experiments with the 2.0 mM H_2_O_2_ treated samples.

### Heat shock at 43°C and 0.5 mM H_2_O_2_ treatment induces increased phosphorylation whereas 2.0 mM H_2_O_2_ treatment induces dephosphorylation of endogenous EhMAPK

**Figure 5:**
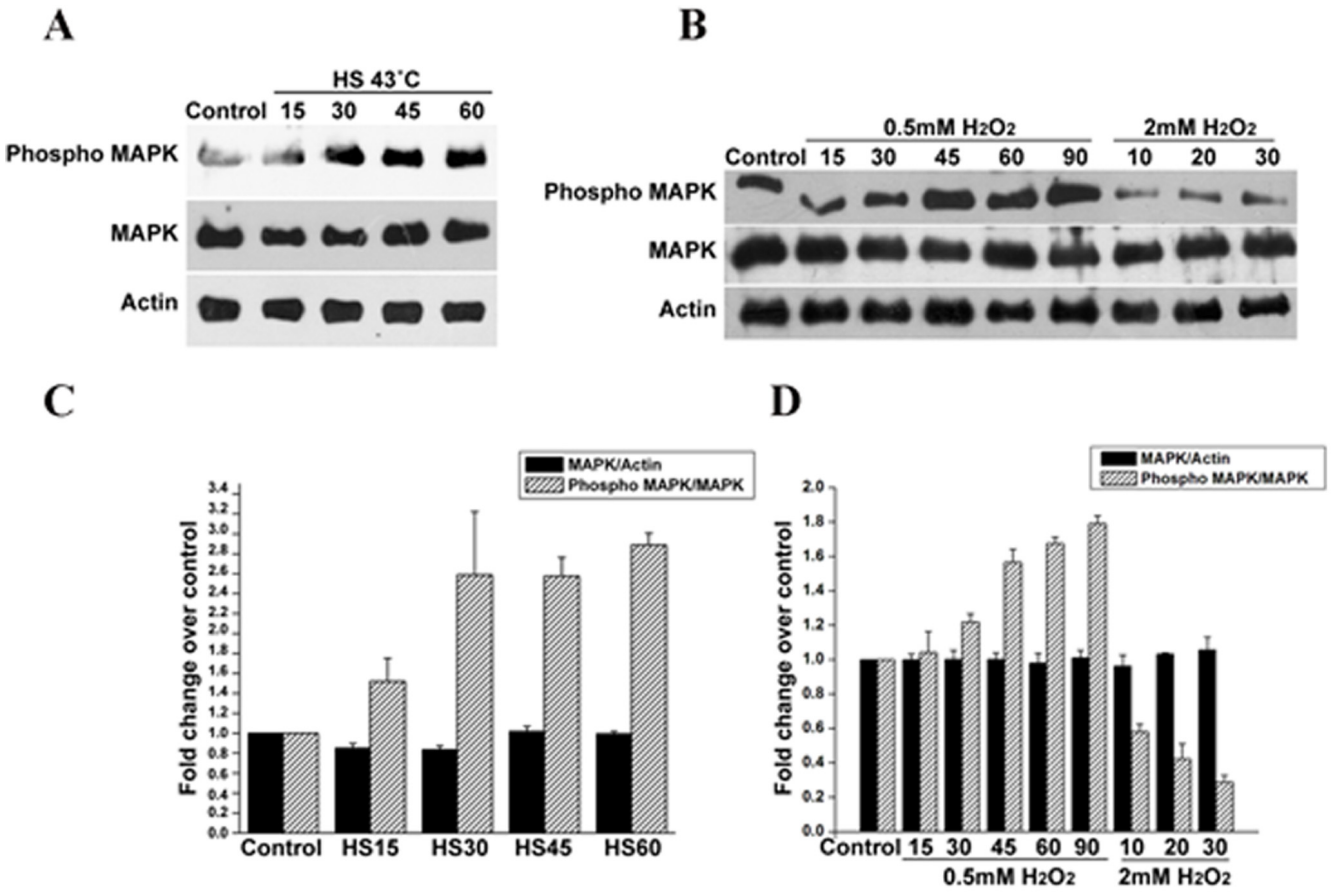
Western immunoblotting of total cell lysates from untreated and treated *E. histolytica* trophozoites. Upper panel: anti phospho ERK1/2, Middle panel: anti EhMAPK, Lower panel: anti actin. (a) Differential phosphorylation of intracellular EhMAPK in response to heat shock. Lane 1: untreated, Lane 2–5: heat shock at 43°C for 15, 30, 45 and 60 min respectively. (b) Differential phosphorylation of intracellular EhMAPK in response to H_2_O_2_ treatment. Lane 1: untreated, Lane 2–6: 0.5 mM H_2_O_2_ for 15, 30, 45, 60 and 90 min respectively, Lane 7–9: 2.0 mM H_2_O_2_ for 10, 20 and 30 min respectively. (c) Densitometric analysis representing the proportions of phosphorylated MAPK to total MAPK and total MAPK to actin in *E. histolytica* trophozoites subjected to heat shock at 43°C for different time periods as a relative change over that of the control untreated trophozoites. (d) Densitometric analysis representing the proportions of phosphorylated MAPK to total MAPK and total MAPK to actin in *E. histolytica* trophozoites treated with either 0.5 mM H_2_O_2_ or 2.0 mM H_2_O_2_ for different time periods as a relative change over that of the control untreated trophozoites. Data represent mean + S.D. of three independent experiments.

The expression profile of EhMAPK at the endogenous protein level matched with that of the mRNA content among untreated, heat shocked and H_2_O_2_ treated samples in all cases of dose and time. But a significant difference was obtained in the phosphorylation status of the protein in response to different stresses. For instance, probing the immunoblots with anti-phospho ERK1/2 antibody which only recognizes the dually phosphorylated form revealed that in response to heat shock at 43°C, the levels of phosphorylated EhMAPK increased as early as 15 min of exposure and became saturated at 60 min exposure (Fig. 5a). Similar was the observation with 0.5 mM H_2_O_2_ where an increase in the phosphorylated EhMAPK was evident within 45 min to 90 min of incubation (Fig. 5b). Moreover the most interesting part was the dephosphorylation of the protein at a lethal dose of 2.0 mM H_2_O_2_. As shown in Fig. 5b an exposure to 2.0 mM H_2_O_2_ induces dephosphorylation of EhMAPK as early as 10 min of exposure and the effect was observed for the 30 min of exposure. The densitometric analysis of the bands corresponding to the endogenous phosphorylated MAPK, total MAPK and actin from the respective western blots gives an estimate of the relative changes of the proportions of phosphorylated MAPK to total MAPK in control untreated trophozoites and those treated with either heat shock at 43°C or 0.5 mM H_2_O_2_ or 2.0 mM H_2_O_2_ for different time points. The phosphorylated MAPK to total MAPK ratio in the trophozoites exposed to heat shock at 43°C for 60 min is ∼2.8 times that of the control untreated trophozoites (Fig. 5c). However in case of H_2_O_2_ treatment an exposure to 0.5 mM H_2_O_2_ for 90 min leads to ∼1.8 times higher and an exposure to 2.0 mM H_2_O_2_ for 30 min leads to 4 times lower intracellular phosphorylated MAPK to total MAPK ratio when compared to the untreated control trophozoites (Fig. 5d). Moreover in order to confirm the specificity of the heterologous anti-phospho ERK 1/2 antibody towards the phosphorylated form of EhMAPK in vivo, a λ phosphatase assay was carried out. Both λ phosphatase-treated and λ phosphatase-untreated TCL from control trophozoites and those exposed to heat shock at 43°C for 60 min were subjected to western blot analysis with anti phospho-ERK1/2, anti-EhMAPK and anti-β actin antibodies (Fig. S1). The absence of bands corresponding to phosphoMAPK in λ phosphatase-treated samples confirmed the specificity of the heterologous anti-phospho ERK 1/2 antibody to the phosphorylated form of the endogenous *E. histolytica* MAPK.

### Differential kinase activities of the endogenous EhMAPK in response to heat shock and H_2_O_2_ treatment

**Figure 6:**
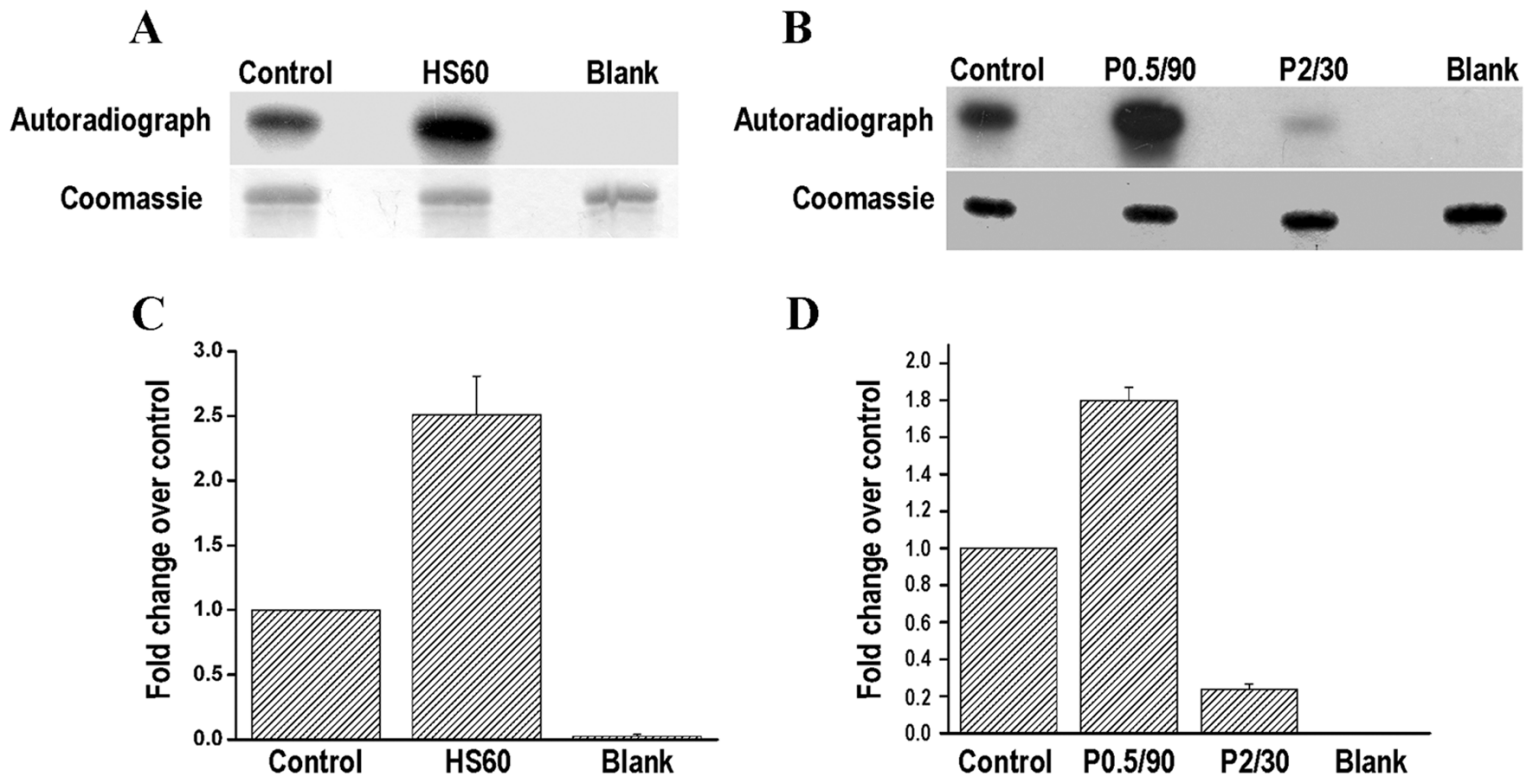
Kinase assay with phosphorylated EhMAPK immunoprecipitated with anti-phospho ERK 1/2 antibody from *E. histolytica* TCL. Lower panel: Coomassie stained gel showing MBP, Upper panel: corresponding autoradiograph. (a) Assay with heat shock samples. Lane 1: untreated, Lane 2: Heat shock at 43°C for 60 min, Lane 3: IP blank (kinase assay in the absence of immunoprecipitated protein). (b) Assay with H_2_O_2_ treated samples. Lane 1: untreated. Lane 2: 0.5 mM H_2_O_2_ for 90 min, Lane 3: 2.0 mM H_2_O_2_ for 30 min, Lane 4: IP blank. (c) Densitometric analysis representing the relative kinase activities of the phosphorylated MAPK from *E. histolytica* trophozoites exposed to heat shock at 43°C for 60 min with respect to that of control untreated trophozoites. (d) Densitometric analysis representing the relative kinase activities of the phosphorylated MAPK from *E. histolytica* trophozoites treated with either 0.5 mM H_2_O_2_ for 90 min or 2.0 mM H_2_O_2_ for 30 min with respect to that of control untreated trophozoites. Data represent mean + S.D. of two different experiments.

**Figure 7:**
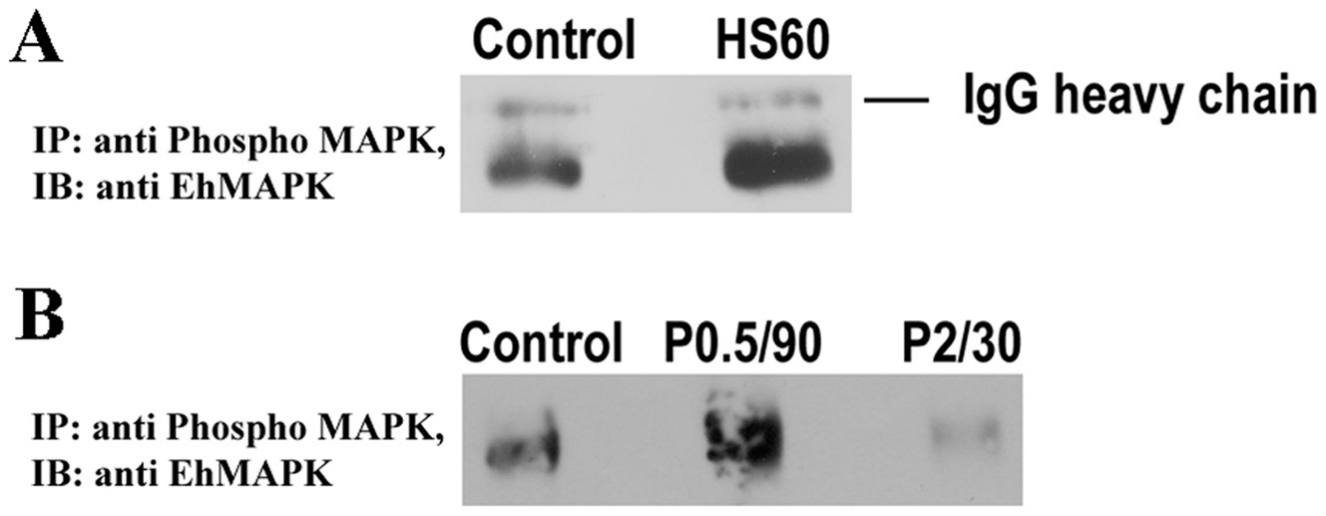
Western immunoblotting of the phosphorylated EhMAPK that is immunoprecipitated with anti-phospho ERK1/2, with anti EhMAPK antibody. Phosphorylated EhMAPK was immunoprecipitated with antiphospho ERK1/2 antibody from the total cell lysate of *E. histolytica* trophozoites either untreated or treated and probed with anti EhMAPK antibody (a) Assay with heat shock samples. Lane 1: untreated, Lane 2: heat shock at 43°C for 60 min. (b) Assay with H_2_O_2_ treated samples. Lane 1: untreated, Lane 2: 0.5 mM H_2_O_2_ for 90 min, Lane 3: 2.0 mM H_2_O_2_ for 30 min.

In the mammalian cells it is well established that the kinase activity of MAPK is associated with the phosphorylation of threonine (T) and tyrosine (Y) residues in the TXY motif of the activation loop [15], [16]. In *E. histolytica* the kinase assays with the purified recombinant EhMAPK also revealed a significant kinase activity associated with only the dually phosphorylated form of the protein which is also recognized by anti-phospho ERK1/2 antibody. So, to investigate the kinase activity of EhMAPK in vivo, kinase assay was performed with the phosphorylated EhMAPK which was immunoprecipitated with anti-phospho ERK1/2 antibody and its ability to phosphorylate MBP was evaluated. As expected a significant kinase activity was found to be associated with the phosphorylated EhMAPK. However when the kinase activities of the immunoprecipitated phospho EhMAPKs from the treated samples were compared to the untreated control samples significant changes were noticed that correlated well with the disparate endogenous levels of phospho EhMAPK as revealed by immunoblots in Fig. 6 and Fig. 7. For instance the increase in the kinase activity of the phospho EhMAPK is clearly evident in case of heat shock at 43°C for 60 min (Fig. 6a) and 0.5 mM H_2_O_2_ treatment for 90 min (Fig. 6b) whereas a decrease in the kinase activity is observed in case of 2.0 mM H_2_O_2_ treatment for 30 min (Fig. 6b). A densitometric analysis of the MBP bands in the autoradiographs and coomassie stained gels represents ∼2.6 times increase in the phosphorylated MBP to total MBP in case of *E. histolytica* trophozoites treated with heat shock at 43°C for 60 min (Fig. 6c), ∼1.8 times increase in case of 0.5 mM H_2_O_2_ treatment for 90 min and ∼4 times decrease in case of 2.0 mM H_2_O_2_ treatment for 30 min (Fig. 6d) when compared to untreated control samples. The decrease in phosphorylation of the EhMAPK is not a non-specific response to 2.0 mM H_2_O_2_ as tyrosine and serine/threonine protein phosphorylation patterns (Fig. S2) show many similarities in general phosphorylation patterns of control and treated samples. Specifically, serine/threonine phosphorylations of proteins of approximate molecular weights of 59 kDa and 90–100 kDA are evident in all samples. Also, proteins with molecular weights between 60 and 25 kDa show copious tyrosine phosphorylation in both 0.5 mM and 2.0 mM peroxide-treated samples. Therefore, it is clear that 2.0 mM H_2_O_2_ treatment did not produce a general protein dephosphorylating effect. Moreover, the western blot analysis of the immunoprecipitated phospho EhMAPK with anti-EhMAPK antibody yielded similar results with a higher proportion of dually phosphorylated MAPK in case of heat shock treatment for 60 min (Fig. 7a) and 0.5 mM H_2_O_2_ treatment for 90 min and a lower proportion in case of 2.0 mM H_2_O_2_ treatment for 30 min (Fig. 7b). However the change in the amount of phosphorylated EhMAPK in response to different treatments when compared to the untreated control could be due to either the change in the amount of the protein getting dually phosphorylated or dephosphorylated or due to the change in the amount of phosphorylation on individual protein molecules if there exist some singly phosphorylated EhMAPK population within *E. histolytica* trophozoites. In the present study we could not discriminate between these two possibilities. Our data are therefore indicative of the increase in the dually phosphorylated EhMAPK in response to heat shock and 0.5 mM H_2_O_2_ treatment and a decrease in response to 2 mM H_2_O_2_ treatment with corresponding increase or decrease in the activity towards its substrate.

## Discussion

*E. histolytica* genome codes for a single typical MAPK that clusters with the ERK7/8 family of MAPKs. Although its sequence has already been characterized in our previous publication [9], its functional implication is largely unexplored till date. Our present study indicates distinct differences in kinase activity of the protein in response to diverse stresses (Fig. 6). Moreover, autophosphorylation of the enzyme has also been linked in the regulation of the kinase activity. In a number of different mammalian cell lines ERK/MAPK activation has often been associated not only with cellular growth and proliferation induced by a number of proliferation-inducing agents [17], [18] but also with different stresses like heat shock, arsenite and oxidative stress [19]–[21]. Our present study emphatically indicates a possible involvement of EhMAPK in heat shock and oxidative stress response in the protozoan. A significant increase in the phospho-EhMAPK species in response to heat shock and 0.5 mM H_2_O_2_ treatment, the stresses which the protozoan can withstand and a corresponding decrease in response to 2.0 mM H_2_O_2_ treatment which is lethal for the parasite point towards a role of EhMAPK in handling of stress conditions by *E. histolytica* in response to a specific stress.

The phylogenetic closeness between protozon MAPKs and mammalian ERK 7/8 has already been noted by Ward et al. [22] in *P. falciparum*, by our group in *E. histolytica* [9] and in *T. gondii* by Lacey et al [23]. The mammalian ERK 7/8 are also phosphorylated on the TEY motif but not by the MEK. Autophosphorylation has been identified as a predominant mechanism of activation of ERK7/8 [24]–[26]. Also, enhanced phosphorylation of ERK8 is observed under DNA-damaging stress conditions including peroxide stress [26]. The fact that the parasite genome lacks MEK, the kinases that usually phosphorylate and activate MAPKs in mammals, is indicative of the presence of some other mechanisms of increasing phosphorylation of EhMAPK during stress survival. Therefore, a distinct possibility exists that an autophosphorylation mechanism may be responsible for the increase in phosphorylation of EhMAPK during heat and 0.5 mM hydrogen peroxide stresses. The *Entamoeba* genome codes for a number of protein phosphatases [27] and a possible involvement of these upstream phosphatases in the maintenance of the phosphorylation status of EhMAPK may also be envisaged.

The kinase assays with the recombinant EhMAPK and subsequent immunoblot analysis using anti phospho tyrosine, anti phospho serine/threonine and anti phospho ERK1/2 antibodies has indeed shown the ability of the protein to undergo autophosphorylation on both threonine as well as tyrosine residues within its TXY activation motif. Moreover one of the interesting features found to be associated with the kinase activity of the recombinant protein towards its substrate MBP was the inability of the singly phosphorylated forms of the protein to carry out substrate phosphorylation as revealed by the kinase assay experiments with the recombinant EhMAPK in the presence of either PTP1B, a tyrosine phosphatase or PP2A, a serine/threonine phosphatase and with the T178A and Y180A mutant EhMAPK proteins.

So we conclude that *E. histolytica* MAPK probably functions as an important signaling molecule for the parasite, associated with the regulation of its survival and death strategies in response to different forms of environmental stress. Moreover, our data reveal that the autophosphorylating ability of EhMAPK may be an important regulatory mechanism. Whether the MAPK phosphatase and dual phosphatase sequences present in *Entamoeba* genome [27] play a role in determining the level of phosphorylation of EhMAPK remains an intriguing question. A study of these upstream elements in relation to EhMAPK activation during stress exposure of *E. histolytica* will shed significant light on the stress response mechanism of the parasite. Based on our data it could be hypothesized that EhMAPK was regulated by a critical balance between its autophosphorylating ability and the activity of the inhibitory phosphatases under normal conditions of cellular homeostasis. Exposure to stress can increase the predominance of the autophosphorylation mechanism resulting in the activation of EhMAPK, thereby enabling the parasite to withstand/overcome the stress while a lethal stress probably results in the predominance of the protein phosphatases leading to the inactivation of MAPK.

## Supporting Information

Figure S1 Western immunoblotting of either lambda phosphatase treated or untreated total cell lysate from E. histolytica trophozoites. Upper panel: anti-phospho ERK1/2, Middle panel: anti-EhMAPK, Lower panel: anti-actin. Lane 1&2: Untreated and heat shocked at 43 degree C for 60 min E. histolytica total cell lysate respectively without lambda phosphatase treatment, Lane 3&4: Untreated and heat shocked at 43 degree C for 60 min E. histolytica total cell lysate respectively with lambda phosphatase treatment.
(0.45 MB TIF)

Figure S2 Phospho Serine/threonine and phospho tyrosine protein phosphorylation patterns in total cell lysates of E. histolytica trophozoites treated with different stresses. A: Western Blot with anti-phospho serine-threonine antibody; B: Western Blot with anti-phospho tyrosine antibody. HS-Heat shock for 60 min at 43 degree C; P0.5/90′–0.5 mM H2O2 for 90 min; P2.0/30′–2.0 mM H2O2 for 30 min.
(4.39 MB TIF)
