# Supplementary figures and images for: EhMAPK, the Mitogen-Activated Protein Kinase from Entamoeba histolytica Is Associated with Cell Survival
Source: PLoS One. 2010 Oct 8;5(10):e13291. doi: 10.1371/journal.pone.0013291 (PMC2951911; doi:10.1371/journal.pone.0013291)

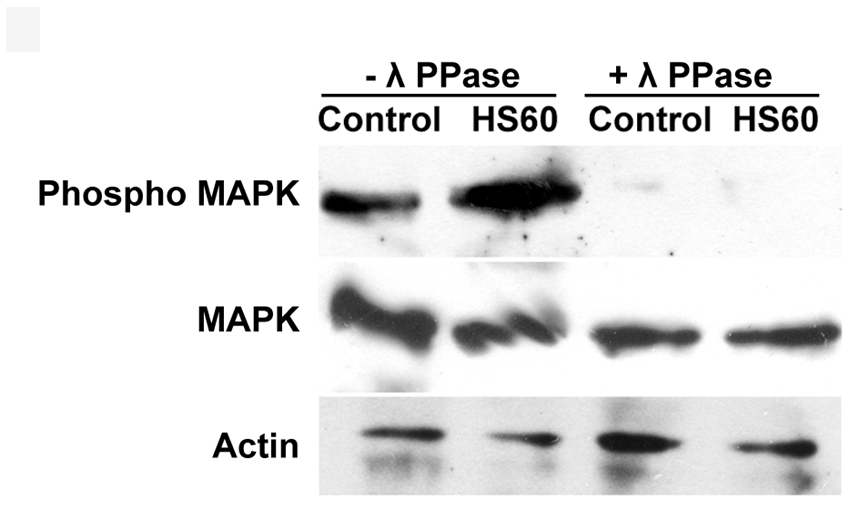

Supplement: Figure S1 — Western immunoblotting of either lambda phosphatase treated or untreated total cell lysate from E. histolytica trophozoites. Upper panel: anti-phospho ERK1/2, Middle panel: anti-EhMAPK, Lower panel: anti-actin. Lane 1&2: Untreated and heat shocked at 43 degree C for 60 min E. histolytica total cell lysate respectively without lambda phosphatase treatment, Lane 3&4: Untreated and heat shocked at 43 degree C for 60 min E. histolytica total cell lysate respectively with lambda phosphatase treatment. (0.45 MB TIF) [file pone.0013291.s001.tif]

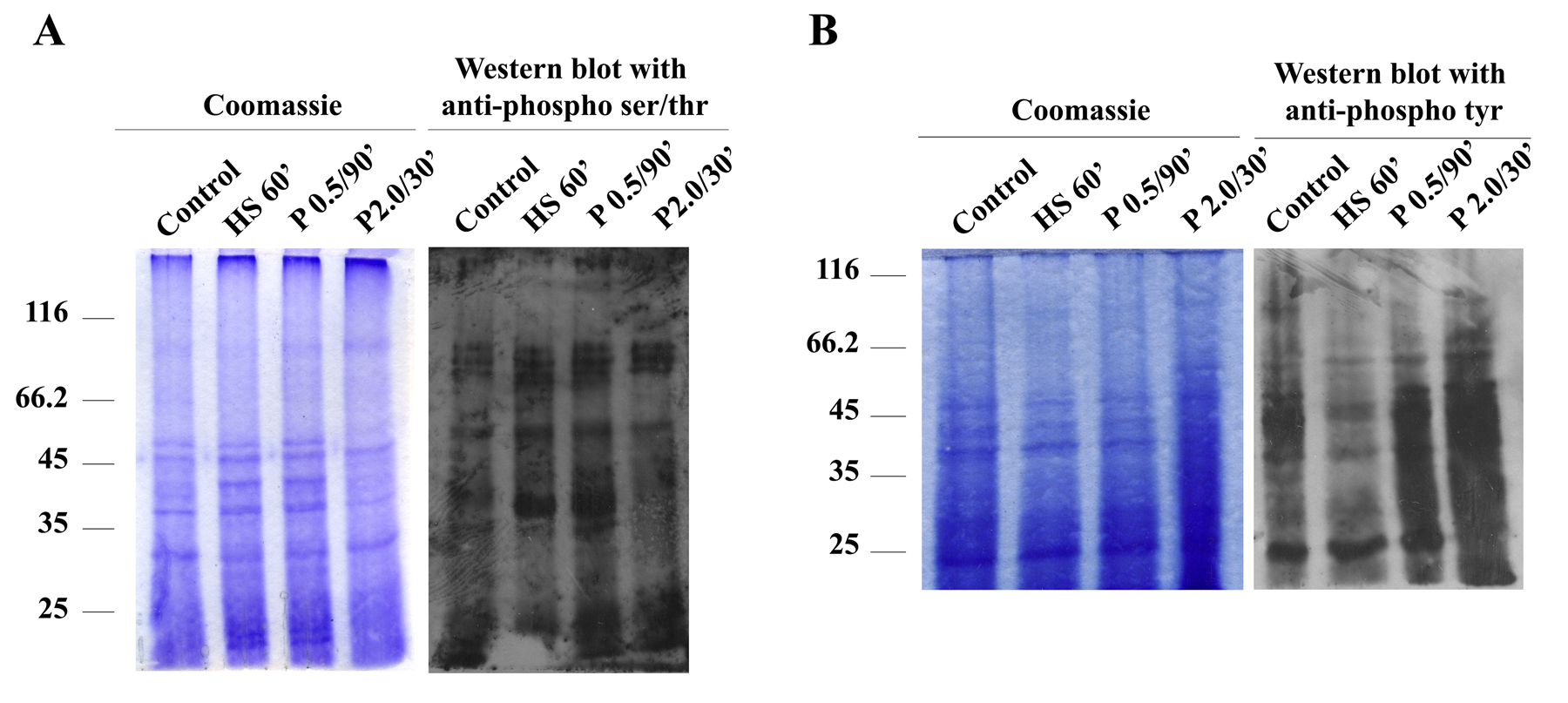

Supplement: Figure S2 — Phospho Serine/threonine and phospho tyrosine protein phosphorylation patterns in total cell lysates of E. histolytica trophozoites treated with different stresses. A: Western Blot with anti-phospho serine-threonine antibody; B: Western Blot with anti-phospho tyrosine antibody. HS-Heat shock for 60 min at 43 degree C; P0.5/90′–0.5 mM H2O2 for 90 min; P2.0/30′–2.0 mM H2O2 for 30 min. (4.39 MB TIF) [file pone.0013291.s002.tif]
